# Polycystic ovary syndrome preceding the diagnosis of acromegaly: a retrospective study in 97 reproductive-aged women

**DOI:** 10.1186/s12958-023-01057-x

**Published:** 2023-02-01

**Authors:** Anamil M. Khiyami, Tahereh Orouji Jokar, Hussein M. Abdallah, Paul A. Gardner, Georgios A. Zenonos, Aaron K. Styer, Pouneh K. Fazeli

**Affiliations:** 1 Neuroendocrinology Unit, Division of Endocrinology and Metabolism, University of Pittsburgh School of Medicine, Pittsburgh, PA USA; 2 College of Medicine, Princess Nourah bint Abdulrahman University, Riyadh, Saudi Arabia; 3 Neuroendocrine Unit, Massachusetts General Hospital, Boston, MA USA; 4 University of Pittsburgh School of Medicine, Pittsburgh, PA USA; 5 Department of Neurological Surgery, University of Pittsburgh School of Medicine, Pittsburgh, PA USA; 6 Beth Israel Deaconess Medical Center and Department of Obstetrics, Gynecology and Reproductive Biology, Harvard Medical School, Boston, MA USA; 7 CCRM Fertility, Boston, MA USA

**Keywords:** Acromegaly, Growth hormone, Hirsutism, IGF-1, Irregular menses, PCOS

## Abstract

**Background:**

Acromegaly is a disease of growth hormone excess that results in enlargement of extremities, abnormal glucose and lipid metabolism, and gonadal disruption. Manifestations of the disease are insidious and typically lead to a diagnostic delay of 7–10 years. Classically the polycystic ovary syndrome (PCOS) phenotype is described in women with irregular menses, clinical or biochemical evidence of androgen excess, and/or multiple ovarian follicles on pelvic ultrasonography. Women with acromegaly may present with some or all of these symptoms. Our objective was to evaluate the prevalence of PCOS in patients with acromegaly and to determine if diagnosis of PCOS results in a delay in diagnosing acromegaly.

**Methods:**

Using patient databases at two academic health centers, we identified 97 premenopausal women aged 18–49 years old presenting with acromegaly. Data were collected regarding pelvic sonography and reproductive history, including the diagnosis of PCOS. Patients carrying the diagnosis of PCOS before their diagnosis of acromegaly were identified and the remaining patients were screened using the Rotterdam criteria to identify additional patients meeting the criteria for PCOS prior to their diagnosis of acromegaly.

**Results:**

Mean age of the population (*n* = 97) at the time of diagnosis of acromegaly was 33.4 ± 7.5 years (SD). Thirty-three percent of patients (*n* = 32) either carried a diagnosis of PCOS or met diagnostic criteria for PCOS before their diagnosis of acromegaly. In the subset of patients in whom data on symptom onset were available, those who met criteria for PCOS were diagnosed with acromegaly a median of 5 years [4, 9] after the onset of symptoms compared to 2 years [0.92, 3] (*p* =  0.006) in the patients who did not meet criteria for PCOS.

**Conclusions:**

Our data demonstrate a high prevalence of signs and symptoms of PCOS in reproductive-aged women with acromegaly and a longer time to diagnosis in women who meet the clinical criteria for PCOS. As screening for acromegaly is relatively simple and done with measurement of a random, non-fasting IGF-1 level that can be drawn at any time during the menstrual cycle, screening patients with PCOS for acromegaly may lessen the delay in diagnosis for reproductive-aged women with this disease.

## Introduction

Acromegaly is a rare disease of growth hormone (GH) excess, most commonly resulting from a pituitary tumor. Acromegaly is characterized by an insidious onset with a mean age of 40 to 45 years at diagnosis [1]. The clinical features of acromegaly, which include coarse facial features, enlarged extremities, disruption of glucose and lipid metabolism, as well as headaches result from the actions of GH excess and local mass effects. Approximately 50% of patients endorse manifestations of hypogonadism at the time of diagnosis [2] and menstrual irregularities as the initial presenting symptom of acromegaly have been reported in approximately 13–21% of premenopausal women [3]. These symptoms are proposed to be secondary to gonadotropin deficiency, hyperprolactinemia, and/or direct effects of GH and insulin-like growth factor (IGF)-1 – a hormone secreted by the liver in response to GH which is responsible for most of GH’s growth-promoting effects – on the ovaries [4].

Prior studies have intimated an association between acromegaly and polycystic ovary syndrome (PCOS) phenotype. Classically the PCOS phenotype is described in women with irregular menses, clinical or biochemical evidence of androgen excess, and/or evidence of multiple ovarian follicles on pelvic ultrasonography [5]. Women with acromegaly may present with some, if not all, of these symptoms [6]. Notably, following treatment for acromegaly, a resolution of polycystic morphology of the ovaries and normalization of menstrual cyclicity has been described, suggesting a direct link between IGF-1 and the PCOS phenotype [4, 6].

The diagnosis of acromegaly is often quite delayed, with an average of 7–10 years between the onset of symptoms and diagnosis [7]. This delay results in larger tumor size at the time of presentation and a lower chance of surgical remission [1]. Screening for acromegaly can be accomplished by serum measurement of IGF-1. A definitively elevated IGF-1 level in a patient with symptoms of GH excess is diagnostic of acromegaly [8]. An IGF-1 level does not need to be obtained while fasting or at any particular time in the menstrual cycle and therefore it is a relatively simple screening test. Using clinical data from three large academic centers with expertise in treating and managing acromegaly, we performed a retrospective study to evaluate the prevalence of PCOS in premenopausal women diagnosed with acromegaly. We hypothesized that a substantial subset of women with acromegaly are diagnosed with PCOS prior to their diagnosis of acromegaly.

## Methods

### Study population

We used a patient database registry at a large academic health system (Mass General Brigham, formerly Partners HealthCare) and a patient registry at the University of Pittsburgh Medical Center (UPMC) to identify women aged 18–49 years old presenting with acromegaly. Patients included from the Mass General Brigham health system presented between 1993 and 2013 and predominantly presented to Massachusetts General Hospital or Brigham and Women’s Hospital. Patients included from UPMC presented to UPMC Presbyterian for surgical resection between 2007 and 2021. All patients were premenopausal women between the ages of 18–49 years. Acromegaly was defined as an IGF-1 level greater than the upper limit of the normal range along with clinical manifestations of the disease such as enlargement of the hands and feet, jaw protrusion, skin tags, sweating, joint pain, or carpal tunnel syndrome.

Once identified through the database, medical records were reviewed for each patient. The presence of metabolic comorbidities including hypertension, prediabetes (defined as 5.7% < hemoglobin A1C < 6.5% or carrying the diagnosis), diabetes mellitus (DM) (hemoglobin A1C > 6.5% or carrying the diagnosis), hyperlipidemia, and body mass index (BMI) were recorded. Data were collected regarding pelvic sonography and reproductive history, including the diagnosis of PCOS. Patients carrying the diagnosis of PCOS before their diagnosis of acromegaly were identified. We then screened the remaining population of patients who did not have a known diagnosis of PCOS and using the Rotterdam criteria [5] (defined as the presence of at least two of the following three symptoms: 1. irregular menses, 2. polycystic ovary morphology on pelvic ultrasonography, and 3. hyperandrogenism, defined as biochemical or clinical evidence of androgen excess, such as the presence of hirsutism or acne), we identified additional patients who met clinical criteria for PCOS before their diagnosis of acromegaly. For patients taking an oral contraceptive pill, we determined if the treatment was used as a method of birth control or for menstrual irregularity. In the subset of patients from UPMC (*n* = 28), we were able to obtain additional data regarding time from onset of initial symptoms of acromegaly to diagnosis and data regarding post-treatment follow-up.

This study was approved by the University of Pittsburgh and Partners HealthCare institutional review boards.

### Statistical analysis

Statistical analyses were performed using JMP Pro 14 (SAS Institute, Carry, NC). Data are presented as the mean ± standard deviation (SD) for continuous variables unless the data were not normally distributed, in which case we report median [interquartile range]. IGF-1 levels are reported as the percent of the upper limit of normal (%ULN). Student’s t-test was used to assess the difference between groups for normally distributed variables and the Wilcoxon signed-rank test for non-normally distributed variables. The Fisher’s exact test was performed to compare categorical variables. A two-sided *p-*value < 0.05 was considered statistically significant.

## Results

### Clinical characteristics of the study population

A total of 97 women were included in the study. The mean age of the population was 33.4 ± 7.5 years [age range: 18–49 years]. The median % upper limit of normal IGF at the time of diagnosis was 177.6 [142.9, 239.6] % and therefore 1.77 times the upper limit of normal (Table 1). For the subset of patients from UPMC (*n* = 28), the median number of years that patients experienced symptoms of acromegaly before a diagnosis was made was 2.5 [1, 6.5] years.

**Table 1:** Clinical characteristics of patients with acromegaly. Mean + SD or *median [interquartile range]

Age	33.4 ± 7.5 years
% Upper limit of normal IGF-1*	177.6 [142.9, 239.6] %
BMI*	26.4 [23.3, 29.7] kg/m^2^
Diabetes Mellitus, % (n)	10.3% (*n* = 10)
Prediabetes, % (n)	28.9% (*n* = 28)
Hypertension, % (n)	18.6% (*n* = 18)
Symptoms at presentation, % (n)	
Irregular menses	58.7% (*n* = 57)
Hirsutism/Hyperandrogenism	32.9% (*n* = 32)
Galactorrhea	18.6% (*n* = 18)
Infertility	14.4% (*n* = 14)
Joint pain	28.9% (*n* = 28)
Carpal tunnel	16.5% (*n* = 16)
Hyperhidrosis	16.5% (*n* = 16)
Snoring	24.7% (*n* = 24)
Macroadenoma, % (n)	68% (*n* = 66)
Microadenoma, % (n)	32% (*n* = 31)

A pituitary macroadenoma, defined as an adenoma > 1 cm in size, was present in 68% of the patients and a microadenoma (pituitary adenoma < 1 cm in size) in 32% of patients. The median BMI of the population was 26.4 [23.3, 29.7] kg/m^2^. Fourteen patients (14.4%) reported a history of infertility before being diagnosed with acromegaly. A total of 36% (*n* = 35) of patients were taking an oral contraceptive pill (OCP) prior to their diagnosis of acromegaly. Of those, nine took the OCP for contraception, and the remaining 26 patients took an OCP due to menstrual irregularity. In the subset of patients from UPMC, 10.7% reported amenorrhea/irregular menses as their initial presenting symptom.

### Prevalence of PCOS in women with acromegaly

Out of the total population, 33% (*n* = 32) met the diagnostic criteria for PCOS before their diagnosis of acromegaly. Of these 32 patients, 13 had been diagnosed with PCOS, while the rest met the diagnostic criteria but did not have a diagnosis indicated in their medical records. In the subset of patients from UPMC, the median number of years from the onset of symptoms of acromegaly to the time of diagnosis of acromegaly in patients who carried a formal diagnosis of PCOS was 4 [1,9] years and the median number of years between diagnosis of PCOS and subsequent diagnosis of acromegaly was 4 [3,8] years.

### Time to diagnosis and follow-up data in acromegalic women with PCOS phenotype versus those without

There was no statistically significant difference between the women who met the criteria for PCOS versus those who did not regarding age at diagnosis of acromegaly, BMI, %ULN IGF-1, prolactin level, or metabolic comorbidities (Table 2). However, in the subset of patients from UPMC (*n* = 28), those meeting criteria for PCOS (*n* = 11) were diagnosed with acromegaly a median of 5 years [4,9] after the onset of symptoms compared to 2 years [0.92,3] (*p* =  0.006) in the patients who did not meet criteria for PCOS. Six of the 11 patients (54.5%) who met criteria for PCOS resumed menses postoperatively and had either clinical or biochemical evidence of resolved hyperandrogenism.

**Table 2:** Comparison between acromegalic patients who carried a diagnosis of PCOS or met diagnostic criteria for PCOS compared to those who did not. Mean + SD or *median [interquartile range]

	With PCOS (***n*** = 32)	Without PCOS (***n*** = 65)	***P***-value
Age (years)	32.5 ± 7.8	33.9 ± 7.3	0.39
%Upper limit of normal IGF-1*	195.4 [151.1, 241.8]	166.7 [141.7, 237.6]	0.37
BMI (kg/m^2^)*	26.9 [24.8, 33.6]	26.2 [22.7, 29.2]	0.16
Prolactin (ng/ml)*	18.3 [7.9, 28.9]	18.3 [9.3, 41.6]	0.47
Impaired glucose tolerance, %	46.9%	35.4%	0.38
Hypertension, %	28.1%	13.8%	0.10

## Discussion

Acromegaly is a disease most commonly due to a pituitary adenoma that results in irreversible changes to physical appearance and systemic complications that may result in premature mortality [2]. The clinical features of this disease are primarily caused by excess IGF-1 production, a hormone secreted in the liver in response to GH; in addition to the hypertrophic effects of IGF-1 on tissue, excess GH results in metabolic complications including insulin resistance. Overall, 60% of the increased mortality observed in this population is thought to be due to cardiovascular disease resulting in part from myocyte hypertrophy and impaired lipid and glucose metabolism [2]. The clinical features of acromegaly are insidious in onset, and the diagnosis is often delayed by an average of 7–10 years [7]; thus, early diagnosis and intervention play a key role in improving the survival of these patients [9].

Approximately 50–75% of all acromegalic patients have gonadal dysfunction at the time of their diagnosis [10]. This is most commonly due to hyperprolactinemia or secondary hypogonadotropic hypogonadism from tumor mass effect. In women, additional mechanisms involving the direct actions of GH and IGF-1 on adrenal and ovarian androgen production [11], as well as hyperandrogenism due to GH mediated-hyperinsulinism, have been proposed [11, 12].

Polycystic ovary syndrome (PCOS) is diagnosed in up to 8–13% of women of reproductive age [13]. One of the most widely used measures for diagnosing this disorder is the Rotterdam criteria which require that patients meet two of the following three criteria: 1) oligo or amenorrhea, 2) androgen excess, or 3) PCO morphology on pelvic ultrasound [5]. The diagnosis of PCOS should only be made after excluding other causes of these criteria, including acromegaly, but given the rarity and likely underdiagnosis of acromegaly, the association between PCOS and acromegaly remains a poorly understood phenomenon. Although IGF-1 has been investigated as a possible etiological factor in PCOS, total IGF-1 levels are not elevated in PCOS compared to controls and therefore, one would not expect IGF-1 levels to be elevated as a result of PCOS alone [14–16].

Several case reports [6, 17–19] and retrospective studies [20] have attempted to further define the relationship between PCOS and acromegaly. In 2007 Kaltsas et al. assessed ovarian morphology in a prospective study of 14 women (21–43 years of age) with active acromegaly [21]. Of the 14 women, 7 met radiographic criteria for PCOS diagnosis and IGF-1 levels were non-significantly higher in the women with PCOS phenotype [21]. Our results demonstrate that approximately 1/3 of premenopausal women diagnosed with acromegaly have signs/symptoms meeting the Rotterdam criteria for PCOS before being diagnosed with acromegaly. Approximately 60% of our cohort had a history of menstrual irregularity, and 30% had a history of hirsutism before being diagnosed with acromegaly. The incidence of PCOS in our population was lower than that of Kaltsas et al. at approximately 50% but higher than the 14% reported by Dogansen et al. [20, 21]; however, given our retrospective study design and lack of ultrasound data in the majority of our patients, these results may be an underestimate of the true prevalence. Importantly, we found that patients who met the diagnostic criteria for PCOS were diagnosed with acromegaly a median of 5 years after the onset of symptoms compared to a median of 2 years in the women who did not meet criteria for PCOS. This suggests that signs/symptoms of PCOS may in fact delay the diagnosis of acromegaly and highlights an important clinical opportunity; screening women who present with signs/symptoms of PCOS for acromegaly may decrease the time to diagnosis in reproductive-aged women with this disease.

In addition to its growth-promoting effects, GH is a lipolytic hormone and has insulin resistance effects [22, 23]. These are adaptive mechanisms that ensure energy availability during states of undernutrition or starvation – states of physiologic stress. In acromegaly, this previously functional adaptation results in a pathologic state of impaired glucose tolerance due to chronically elevated GH levels. In turn, hyperinsulinism is a likely cause of increased androgen biosynthesis from the ovary [24–27], and also possibly the adrenal [28].

Levels of IGF-1, which is a hormone produced by the liver in response to GH stimulation, are also increased in acromegaly and IGF-1 may have direct effects on the ovaries [29]. There are IGF-1 receptors on thecal cells and IGF-1 may directly stimulate the activity of 17-hydroxylase and 17,20 lyase upregulating androgen production [30]. Therefore, both GH-induced hyperinsulinism and elevated IGF-1 levels are likely contributors to the hyperandrogenic state in acromegaly resulting in clinical signs/symptoms quite similar to those of PCOS [30].

Irregular menstrual cycles are a common sign/symptom in women with acromegaly. In the subset of patients from UPMC, this was the presenting symptom in over 10% of this cohort of reproductive-aged women diagnosed with acromegaly. Grynberg et al. assessed 38 women with acromegaly to clarify the cause of their gonadal dysfunction [4]. Approximately 80% of cases were attributed to hyperprolactinemia, hypogonadotropic hypogonadism, or a mixed cause [4]. In the remaining patients, gonadal dysfunction could only be attributed to perturbation of the GH/IGF-1 axis and two had polycystic ovary syndrome phenotype, which resolved with normalization of GH and IGF-1 levels [4]. In our study, 54.5% of patients who met criteria for PCOS had resumption of menses and either clinical or biochemical evidence of resolved hyperandrogenism post-operatively. Therefore, acromegaly should be an important diagnostic consideration in patients presenting with clinical signs/symptoms of PCOS and our data highlight both the high prevalence of PCOS in reproductive-aged women with acromegaly and a potential increased time to diagnosis in women with acromegaly who meet the diagnostic criteria for PCOS.

Limitations of our study include the fact that it is a retrospective study reliant on clinical medical records. The reliance on clinical data and the fact that a limited number of patients had documented pelvic ultrasonography, may possibly have led to an underestimate of the prevalence of PCOS in this population of women with acromegaly. We therefore anticipate that more complete data would have likely strengthened our findings.

## Conclusions

In conclusion, acromegaly is a disease with an indolent evolution, and the average period between symptom onset and diagnosis is prolonged. Screening for acromegaly is relatively simple and done with measurement of a random, non-fasting IGF-1 level that can be drawn at any time during the menstrual cycle. Given the high prevalence of clinical signs and symptoms of PCOS in this population, our data suggest that screening patients with PCOS symptoms for acromegaly may lessen the delay in diagnosis for reproductive-aged women with this disease.

## Data Availability

The datasets (deidentified) used and/or analyzed in the current study are available from the corresponding author on reasonable request.
